# Effect of growth hormone adjuvant treatment on oocyte retrieval in patients with poor ovarian response and no viable embryos in previous *in vitro* fertilization (IVF) cycles

**DOI:** 10.7717/peerj.21000

**Published:** 2026-03-16

**Authors:** Liang Gao, Yuting Zhang, Mengyun Li, Jingyu Zhang, Jianjun Zhou

**Affiliations:** 1 Drum Tower Clinic Medical College of Nanjing Medical University, Nanjing, China; 2 The Affiliated Taizhou People’s Hospital of Nanjing Medical University, Taizhou, China; 3 Nanjing Drum Tower Hospital, Affiliated Hospital of Medical School, Nanjing University, Nanjing, China

**Keywords:** Growth hormone, Poor ovarian response, Transferable embryo, Luteinizing hormone

## Abstract

**Background:**

This study aimed to evaluate the efficacy of growth hormone (GH) as an adjuvant treatment for enhancing embryo quantity in patients with poor ovarian response (POR) who previously had no viable embryos in in vitro fertilization (IVF) cycles.

**Methods:**

A total of 86 POR patients with no viable embryos in their first IVF cycles were enrolled. Among them, 43 patients in the study group received GH adjuvant treatment during their second IVF cycle, while the remaining 43 patients in the control group did not receive GH. Clinical characteristics were compared between the GH+ and GH− cycles within the study group, and further comparisons were made between the study and control groups regarding clinical characteristics and IVF outcomes.

**Results:**

Self-controlled analysis showed that GH adjuvant treatment significantly increased the number of oocytes retrieved (*P* = 0.010), bipronuclear (2PN) embryos (*P* = 0.007), and transferable embryos (*P* < 0.001) in the study group. Compared to the control group, study group had a higher number of oocytes retrieved (*P* = 0.029) and a higher luteinizing hormone (LH) level on the HCG trigger day (*P* = 0.049). Multivariate logistic regression analysis identified LH level on the HCG trigger day in the GH cycle as an independent factor associated with increased oocyte retrieval (OR: 1.149, 95% CI [1.007–1.310], *P* = 0.038).

**Conclusion:**

GH adjuvant treatment may improve the number of oocytes retrieved in POR patients who previously had no viable embryos in IVF cycles.

## Introduction

Poor ovarian response (POR) is one of the most challenging issues affecting *in vitro* fertilization (IVF) and intracytoplasmic sperm injection (ICSI) outcomes (Ferraretti et al., 2011); approximately 4.7–20% of patients cancel their IVF cycle due to POR. The absence of viable embryos is a key factor that can lead to the cancellation of an IVF cycle (Humaidan et al., 2017; Giovanale et al., 2015). The incidence of POR varies between 9% and 24%, with a trend of increasing prevalence annually (Drakopoulos et al., 2020). The precise etiology of POR is not fully understood. Some recognized causes include age-related follicular depletion, advanced endometriosis, and chromosomal and genetic alterations (Blumenfeld, 2020; Nie et al., 2024; Nelson, 2009). Age is a significant factor, as the natural decline in antral follicle count reduces both oocyte quantity and quality. This culminates in fewer viable embryos for transfer, often leading to cycle cancellation and contributing substantially to diminished IVF success rates.

Growth hormone (GH), a polypeptide hormone primarily secreted by the pituitary gland, is essential for stimulating skeletal and visceral growth, enhancing protein synthesis, and influencing lipid and mineral metabolism (Strobl & Thomas, 1994). Research suggests that GH serves as a pivotal regulator of ovarian steroidogenesis, follicular development, and oocyte maturation. However, the exact cellular and molecular mechanisms through which GH impacts the reproductive system have not been fully elucidated. Studies show that GH may be used as an adjuvant therapy in IVF to improve embryo quality (Dosouto et al., 2019; Zhang et al., 2020; Zhu et al., 2023).

Research has shown that GH stimulates the production and release of insulin-like growth factor 1 (IGF-1) in the ovaries, a critical process for the development of follicles from a gonadotropin-insensitive to a gonadotropin-sensitive phase (Dosouto et al., 2019; Hull & Harvey, 2014; Nasioudis et al., 2019). IGF-1 functions through both paracrine and autocrine mechanisms to boost steroidogenesis and encourage the multiplication of granulosa cells (Kucera et al., 2018) Additionally, GH can directly enhance the nuclear maturation of human oocytes by interacting with GH receptors (GHRs) located within the oocytes, thereby enhancing oocyte quality (Xu, Hao & Gao, 2019). Over the last thirty years, GH has been integrated into assisted reproductive technology (ART) (Chang et al., 2022), prompting numerous clinical studies to investigate its potential impact. Laboratory results and retrospective analyses suggest that administering GH before gonadotropin (Gn) therapy could positively affect the quantity of retrieved oocytes (Kucuk, Kozinoglu & Kaba, 2008). Similarly, the efficacy of GH in enhancing embryo quality among women with POR has been substantiated (Du et al., 2016). Some studies have indicated that the use of GH could improve clinical outcomes, including pregnancy and live birth rates (Yovich & Stanger, 2010; Liu et al., 2024; Ho et al., 2017). Systematic reviews and meta-analyses have corroborated the advantageous effects of GH (Hart, Rombauts & Norman, 2017; Liu et al., 2025; Lin et al., 2023).

Despite these positive findings, some studies have failed to demonstrate a significant improvement in outcomes. A randomized controlled double-blind trial focusing on GH administration during IVF in women with normal ovulation revealed no enhancement in ovarian response or embryo quality (Blumenfeld, 2020). Similarly, a randomized placebo-controlled trial by Norman et al. (2019) failed to demonstrate a significant increase in live birth rates with GH supplementation in poor ovarian responders, despite improvements in intermediate outcomes. The lack of significant improvement in some studies could be influenced by various factors including heterogeneity in patient inclusion criteria, differences in study design (such as variations in GH administration and ovarian stimulation protocols), limited statistical power, and small sample sizes. Consequently, the impact of GH on ovarian response and embryo quality in the poor ovarian response patients who had no viable embryo in their previous IVF cycles remains uncertain. The objective of this retrospective study was to investigate whether GH adjuvant treatment is advantageous for these patients.

## Materials and Methods

### Study population

This retrospective observational study was conducted at the reproductive center of Nanjing Drum Tower Hospital, the Affiliated Hospital of Nanjing University Medical School, from August 2016 to June 2019. The study protocol was approved by the ethics committee of Nanjing Drum Tower Hospital (2021-183-01). Due to the retrospective nature of the medical record analysis, informed consent requirements were waived.

POR was diagnosed according to the Bologna criteria (Ferraretti et al., 2011), defined as the presence of at least two of the following three features: (i) advanced maternal age (≥40 years) or the presence of other risk factors for poor ovarian response; (ii) a previous episode of poor ovarian response, defined as ≤3 oocytes retrieved following a conventional ovarian stimulation protocol; (iii) decreased ovarian reserve, defined as an antral follicle count (AFC) <5–7 follicles or an anti-Müllerian hormone (AMH) level <0.5–1.1 µg/L. In addition to meeting the diagnostic criteria for POR, all included patients had experienced at least one previous IVF cycle resulting in no transferable embryos. The exclusion criteria were as follows: (i) karyotype abnormalities in one or both partners, (ii) patients with abnormal uterine cavities and bodies, including thin endometrium (<7 mm), endometrial polyp, intrauterine adhesions, uterine deformity, and submucosal myoma or adenomyosis, (iii) male partners with azoospermia and severe oligospermia. A blastocyst is assessed according to its trophoblast cell grade, inner cell mass grade, and developmental stage. It is referred to as a transferable embryo when its grade is better than 4CC.

All patients included in the study had adverse outcomes in previous IVF cycles and were offered GH as an adjuvant treatment by their attending clinicians (independent of the researchers) in the following cycle based on clinical judgment from previous cycles. The patients served as their own controls.

### Clinical management

There was no strict limitation on the ovarian stimulation protocol, as studies have shown that the type of stimulation does not independently influence embryo quality (Polyzos et al., 2014). Four conventional controlled ovarian hyperstimulation (COH) protocols including Gonadotropin-releasing hormone (GnRH) agonist protocol, GnRH antagonist protocol, mild stimulation protocol, and progestin primed ovarian stimulation protocol (PPOS) were included in this study. In the GnRH agonist protocol, long-acting triptorelin acetate (*e.g.*, 0.94 mg or 1.875 mg administered once) or short-acting triptorelin acetate (0.1 mg or 0.05 mg daily) was injected during the midluteal phase of the previous menstrual cycle. Gn was administered 14–35 days later. The dosage of Gn was adjusted based on the ovarian response, which was monitored through transvaginal ultrasonography and serum hormone levels. In the GnRH antagonist protocol, Gn was administered from the second day of menstrual cycle. The dosage was adjusted according to the ovarian response and serum hormone levels. GnRH antagonist was given 0.25 mg daily when the dominant follicles reached a diameter of 14 mm until the day of hCG trigger. In the mild stimulation protocol, a daily dose of 50 mg of Clomiphene citrate (Fertilan; Codal Synto Ltd., Cyprus) was given from Day 2 of the menstrual cycle and/or 75 international unit (IU) human menopausal gonadotropin (HMG) (Lebaode; Lizhu Pharmaceutical Co, China). In the PPOS protocol, 150–300 IU Gn and 10 mg of medroxyprogesterone acetate (MPA) were administered daily from Day 2 of the menstrual cycle until the day of the human chorionic gonadotropin (hCG) trigger. When the dominant follicles reached a diameter of 18 mm, 10,000 IU of hCG was administered. Transvaginal oocyte retrieval was performed 36 h after the administration of hCG. GH was administered *via* hypodermic injection at a daily dosage of 2 IU starting in the follicular or luteal phase of the preceding cycle and continuing for 2–5 weeks until the hCG trigger. This dosage represents the equivalent GH regimen routinely used in the study center for POR patients. Embryos were cultured sequentially in Vitrolife G1 and G2 media at 37 °C in an ASTEC incubator (Japan) under low-oxygen conditions (5% O_2_, 5% CO_2_, and 90% N_2_). Embryos were cultured in G1 medium until day 3 and then transferred to G2 medium for continued culture until the blastocyst stage. Embryo culture was performed using Vitrolife G1 culture medium. All embryos were cultured at 37 °C in a Japan ASTEC incubator under a controlled low-oxygen atmosphere consisting of 5% O_2_, 5% CO_2_, and 90% N_2_. Assessment of embryos was performed based on the international morphological system including the Peter scoring system for cleavage embryos and the Gardner scoring system for blastocysts.

### Statistical analysis

Data analysis was performed using SPSS version 26.0. Quantitative variables were assessed for normality using the Shapiro–Wilk test and for homogeneity of variance using Levene’s test. Normally distributed continuous variables with equal variances were presented as mean ± standard deviation and compared between groups using independent-samples Student’s t-tests. When normality or homogeneity of variance assumptions were violated, the Mann–Whitney U test was applied instead. Categorical variables were expressed as frequencies and percentages and compared using the chi-square test or Fisher’s exact test when appropriate. Binary logistic regression analysis was performed to calculate odds ratios (ORs) with 95% confidence intervals (CIs). Receiver operating characteristic (ROC) curves were constructed to evaluate the discriminatory performance of each variable. A *P* value <0.05 was considered statistically significant.

As this was a retrospective observational study including all eligible patients during the study period, no priori sample size calculation was performed. A *post-hoc* power analysis was conducted for the primary outcome—the number of oocytes retrieved. Based on the observed values (GH− cycle: 0; GH+ cycle: 0.81 ± 0.96), the calculated effect size (Cohen’s *d* = 0.92) indicated a high effect magnitude. Using this effect size with *α* = 0.05 (two-tailed), the statistical power of the study was greater than 90%, demonstrating that the study had sufficient power to detect the observed difference in oocyte yield between the two cycles.

## Results

This study included 86 POR patients with no transferable embryos in their first IVF cycles. Forty-three (43) patients received GH adjuvant treatment in the second cycle, forming the study group. The first IVF cycle of these 43 patients served as their own control. Additionally, 43 other POR patients who did not receive GH adjuvant treatment in the second IVF cycle formed the control group. They were matched to the study group based on similar clinical data from their first cycle.

### Comparison of clinical characteristics between GH− and GH+ cycles in the study group

Clinical characteristics of the two cycles in both the GH− and GH+ groups are summarized in Table 1. Statistically significant differences (*P* < 0.05) were observed for female age, male age, progesterone level on the trigger day, the number of 2PN embryos, the number of transferable embryos, and the transferable embryo cycle rate. No statistically significant differences were observed for the other indicators.

### Comparison of clinical characteristics between the study and control groups

To further clarify the impact of GH adjuvant treatment on patients with POR, the relevant clinical characteristics were compared between the study group and the control group. In their first cycle, there were no statistically significant differences in basic data between the two groups (Table 2). Subsequently, the clinical characteristics of the second cycle were compared between the study group and control group (Table 3). The results indicated that the luteinizing hormone (LH) level on the HCG trigger day was lower in the study group compared to the control group (*P* = 0.049), while the number of oocytes retrieved was higher than that in the control group (*P* = 0.029). No other indicators showed statistically significant differences, suggesting that GH adjuvant treatment may increase the number of oocytes retrieved.

**Table 1:** Clinical characteristics of patients between the GH (+) cycle and GH (−) cycle in the study group.

Characteristics	The GH-cycle (*n* = 43)	The GH+ cycle (*n* = 43)	*P* value
Female age (years)	40.23 ± 4.80	40.58 ± 4.80	<0.001
Male age (years)	41.47 ± 5.29	41.72 ± 5.25	<0.001
Basal LH (IU/L)	5.29 ± 2.56	5.29 ± 2.56	–
Basal FSH (IU/L)	11.65 ± 7.36	11.65 ± 7.36	–
AFC (n)	5.28 ± 2.07	5.28 ± 2.07	–
Protocol of COS			
Progestin Primed Ovarian Stimulation (PPOS) protocol	5	3	0.171
GnRH agonist long protocol	4	0	
GnRH antagonist protocol	2	2	
Mild stimulation protocol	32	38	
Duration of COS (day)	8.74 ± 3.44	8.56 ± 2.18	0.773
Endometrium thickness on hCG trigger day (mm)	7.23 ± 2.30	6.88 ± 1.90	0.444
E2 level on hCG trigger day / Number of oocytes retrieved (n)	546.77 ± 641.10	642.99 ± 329.10	0.384
LH level on hCG trigger day (IU/L)	9.50 ± 6.93	8.24 ± 4.88	0.332
FSH level on hCG trigger day (IU/L)	19.77 ± 9.67	17.16 ± 7.44	0.164
P level on hCG trigger day (ng/ml)	0.69 ± 0.31	0.88 ± 0.53	0.046
Number of oocytes retrieved (n)	1.65 ± 1.00	2.58 ± 2.10	0.010
Number of 2PN (n)	0.93 ± 0.91	1.74 ± 1.71	0.007
Number of transferable embryos (n)	0	0.81 ± 0.96	<0.001
Number of cycles with embryos available for transfer (%)	0% (0/43)	53.5% (23/43)	<0.001

**Notes.**

Data are presented as mean ± standard deviation (SD) or number (%). GH (+): cycles with growth hormone adjuvant treatment; GH (−): cycles without growth hormone treatment. *P* < 0.05 was considered statistically significant.

**Table 2:** Clinical characteristics of the first IVF cycle between the control group and the study group.

Characteristics	The control group (*n* = 43)	The study group (*n* = 43)	*P* value
Female age (years)	40.65 ± 5.12	40.23 ± 4.80	0.696
≥40 years (n)	67.4% (29/43)	74.4% (32/43)	0.476
<40 years (n)	32.6% (14/43)	25.6% (11/43)	
Male age (years)	41.70 ± 7.65	41.47 ± 5.29	0.872
Basal LH (IU/L)	6.15 ± 3.99	5.29 ± 2.56	0.238
Basal FSH (IU/L)	12.94 ± 7.65	11.65 ± 7.36	0.428
AFC (n)	5.49 ± 1.79	5.28 ± 2.07	0.616
AFC<7 (n)	86.0% (37/43)	74.4% (32/43)	0.176
AFC≥7 (n)	14.0% (6/43)	25.6% (11/43)	
Protocol of COS			
Progestin Primed Ovarian Stimulation (PPOS) protocol	6	5	0.765
GnRH agonist long protocol	2	4	
GnRH antagonist protocol	1	2	
Mild stimulation protocol	34	32	
Duration of COS (day)	8.88 ± 3.20	8.74 ± 3.44	0.846
Endometrium thickness on hCG trigger day (mm)	7.40 ± 2.08	7.23 ± 2.30	0.866
E2 level on hCG trigger day / Number of oocytes retrieved	569.04 ± 251.63	638.71 ± 326.12	0.271
LH level on hCG trigger day (IU/L)	10.80 ± 8.86	9.50 ± 6.93	0.451
FSH level on hCG trigger day (IU/L)	19.98 ± 10.24	19.77 ± 9.67	0.922
P level on hCG trigger day (ng/ml)	0.75 ± 0.52	0.69 ± 0.31	0.518
Number of oocytes retrieved (n)	1.47 ± 0.88	1.65 ± 1.00	0.378
Number of 2PN (n)	0.67 ± 0.92	0.93 ± 0.91	0.191
Number of transferable embryos (n)	0	0	–
Number of cycles with embryos available for transfer (%)	0	0	–

**Notes.**

Values are expressed as mean ± standard deviation (SD) or number (%). Control group: patients without growth hormone adjuvant treatment; study group: patients who received growth hormone adjuvant treatment. *P* < 0.05 was considered statistically significant.

**Table 3:** The clinical characteristics and IVF outcomes in the second IVF cycle between the control and study groups.

Characteristics	The control group (*n* = 43)	The study group (*n* = 43)	*P* value
Female age (years)	41.00 ± 5.04	40.58 ± 4.80	0.694
≥40 years (n)	67.4% (29/43)	76.7% (33/43)	0.336
<40 years (n)	32.6% (14/43)	23.3% (10/43)	
Male age (years)	42.00 ± 7.60	41.72 ± 5.25	0.837
Basal LH (IU/L)	6.15 ± 3.99	5.29 ± 2.56	0.238
Basal FSH (IU/L)	12.94 ± 7.65	11.65 ± 7.36	0.428
AFC (n)	4.49 ± 1.79	5.28 ± 2.07	0.630
AFC<7, n (%)	86.0% (37/43)	74.4% (32/43)	0.176
AFC≥7, n (%)	14.0% (6/43)	25.6% (11/43)	
Protocol of COS			
Progestin Primed Ovarian Stimulation (PPOS) protocol	4	3	0.788
GnRH agonist long protocol	0	0	
GnRH antagonist protocol	1	2	
Mild stimulation protocol	38	38	
Duration of COS (day)	8.86 ± 3.48	8.56 ± 2.18	0.633
Endometrium thickness on hCG trigger day (mm)	7.06 ± 3.76	6.88 ± 1.90	0.776
E2 level on hCG trigger day / Number of oocytes retrieved	577.54 ± 245.39	546.27 ± 296.54	0.596
LH level on hCG trigger day (IU/L)	10.43 ± 5.28	8.24 ± 4.88	0.049
FSH level on hCG trigger day (IU/L)	18.45 ± 8.61	17.16 ± 7.44	0.458
P level on hCG trigger day (ng/ml)	0.78 ± 0.43	0.88 ± 0.53	0.340
Number of oocytes retrieved (n)	1.74 ± 1.31	2.58 ± 2.10	0.029
Number of 2PN (n)	1.26 ± 1.11	1.63 ± 1.51	0.127
Number of transferable embryos (n)	0.88 ± 1.03	0.81 ± 0.96	0.746
Number of cycles with embryos available for transfer (%)	55.8% (24/43)	53.5% (23/43)	0.829

**Notes.**

Values are expressed as mean ± standard deviation (SD) or number (%), unless otherwise indicated. Control group: patients without growth hormone adjuvant treatment; study group: patients who received growth hormone adjuvant treatment. *P* < 0.05 was considered statistically significant.

### Binary logistic analysis of factors associated with increased oocyte retrieval

**Figure 1:**
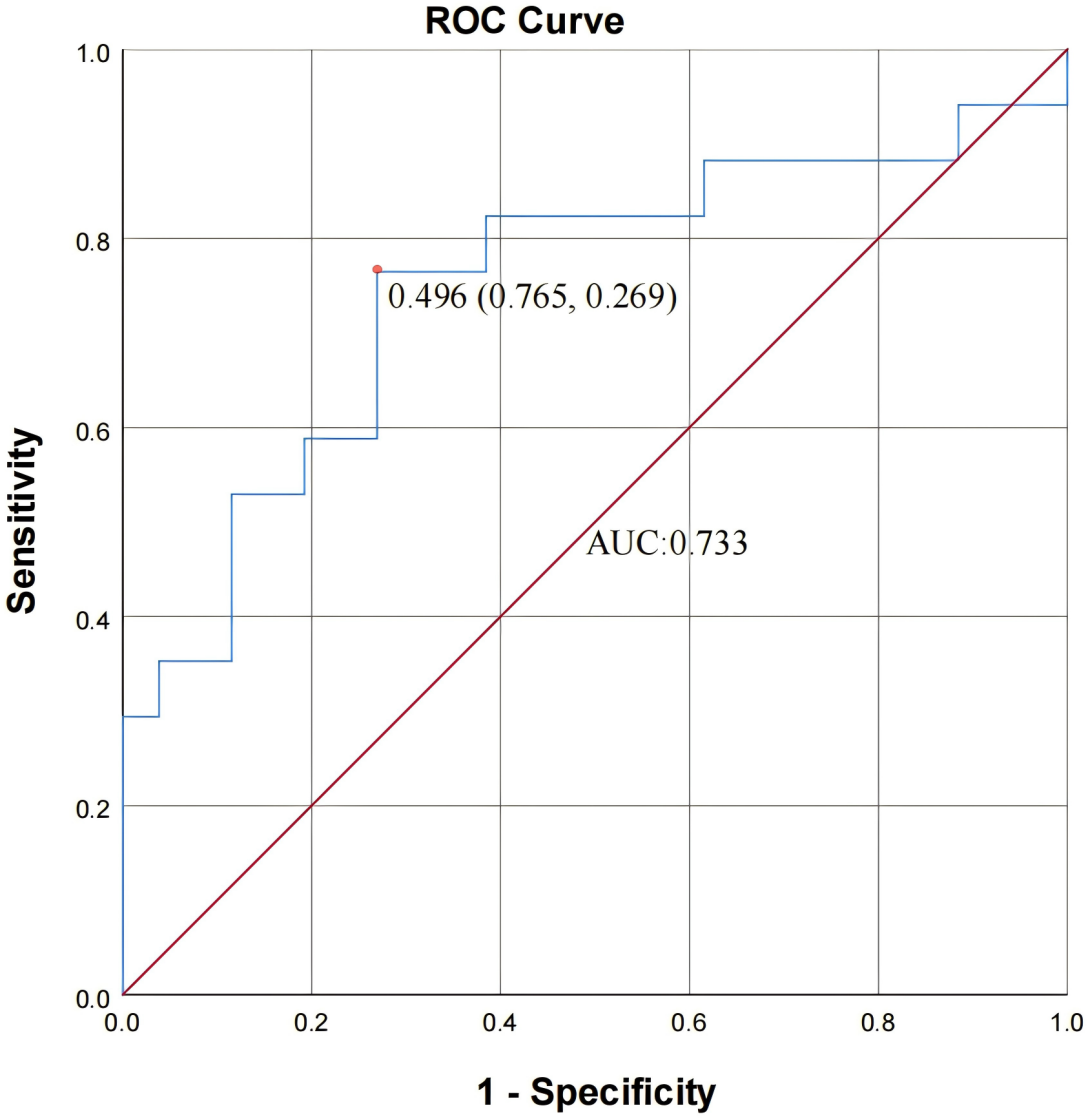
Receiver operating characteristic (ROC) curve of LH levels on the hCG trigger day in the GH(−) cycle for predicting increased oocyte retrieval following GH adjuvant treatment. The area under the ROC curve (AUC) was 0.733 (95% CI [0.587–0.911]), indicating a moderate predictive value of LH level on the hCG trigger day for identifying patients who showed an increased number of oocytes retrieved after GH adjuvant therapy. At the cutoff value of LH = 8.755 IU/L, the sensitivity and specificity were 76.5% and 73.1 %, respectively.

To further investigate which POR patients benefit from GH adjunctive therapy in terms of increased oocyte retrieval, patients in the study group were categorized based on an increase (or not) in the number of oocytes retrieved in their second cycle, forming an increased group and a non-increased group. Clinical characteristics were analyzed from their first cycle, and univariate analysis revealed statistically significant differences in progesterone level (*P* = 0.028) and LH level on the HCG trigger day (*P* = 0.004), while no other differences reached statistical significance (Table 4). To explore the factors associated with increased oocyte retrieval after GH adjuvant treatment, binary logistic regression analyses were performed. In univariate analysis, the LH level on the hCG trigger day was significantly associated with increased oocyte retrieval (OR = 1.169, 95% CI [1.029–1.327], *P* = 0.016), indicating that higher LH values were related to a greater likelihood of oocyte increase. Progesterone level on the hCG day showed a borderline association (OR = 6.658, 95% CI [0.780–56.819], *P* = 0.083), but it lost significance in the multivariate model. Other factors, including female age, male age, basal hormone levels, AFC, controlled ovarian stimulation (COS) protocol, endometrial thickness, and the E2/AFC ratio, were not significant predictors. Multivariate logistic regression further confirmed that LH level on the hCG trigger day remained the only independent predictor (OR = 1.149, 95% CI [1.007–1.310], *P* = 0.038; Table 5). An ROC analysis comparing LH level on the HCG trigger day in the first cycle of the study group between the number of oocytes retrieved from the increased and not increased group was conducted. The area under ROC curve (AUC) for LH level was 0.733 (95% CI [0.587–0.911]; Fig. 1). The optimal cut-off value proposed by the ROC analysis for LH level was 8.755 IU/L.

**Table 4:** Clinical characteristics in GH(−) cycles between patients with and without increased oocyte retrieval in the GH(+) cycle.

Characteristics	The number of oocytes retrieved increased group (*n* = 17)	The number of oocytes retrieved not increased group (*n* = 26)	*P* value
Female age (years)	40.12 ± 4.08	40.31 ± 5.29	0.774
Male age (years)	42.18 ± 5.10	40.96 ± 5.46	0.456
Basal LH (IU/L)	5.13 ± 2.14	5.40 ± 2.85	0.488
Basal FSH (IU/L)	10.43 ± 9.50	12.36 ± 5.71	0.398
AFC (n)	4.88 ± 1.93	5.40 ± 2.86	0.366
Protocol of COS			
Progestin Primed Ovarian Stimulation (PPOS) protocol	2	3	0.594
GnRH agonist long protocol	1	3	
GnRH antagonist protocol	0	2	
Mild stimulation protocol	14	18	
Duration of COS (day)	8.35 ± 2.81	8.69 ± 1.69	0.613
Endometrium thickness on hCG trigger day (mm)	6.97 ± 2.54	7.33 ± 2.21	0.619
E2 level on hCG trigger day / Number of oocytes retrieved	272.51 ± 234.63	427.08 ± 784.06	0.424
LH level on hCG trigger day (IU/L)	13.56 ± 8.74	7.20 ± 4.56	0.004
FSH level on hCG trigger day (IU/L)	18.83 ± 12.01	19.76 ± 8.19	0.759
P level on hCG trigger day (ng/ml)	0.85 ± 0.35	0.62 ± 0.30	0.028
Number of oocytes retrieved (n)	2.00 ± 3.34	3.00 ± 4.22	0.404
Number of oocytes retrieved/AFC	0.44 ± 0.58	0.91 ± 1.84	0.306

**Notes.**

Values are expressed as mean ± standard deviation (SD) or number (%). GH(+), cycles with growth hormone adjuvant treatment; GH(−): cycles without growth hormone adjuvant treatment. Comparisons were performed using Student’s *t*-test or *χ*^2^ test, as appropriate. *P* < 0.05 was considered statistically significant.

**Table 5:** Binomial logistic regression analysis of factors in the GH(−) cycle associated with increased oocyte retrieval after GH adjuvant treatment.

Variables	Univariate analysis			Multivariate analysis		
	OR	95% CI	*P*-Value	OR	95% CI	*P*-Value
Female age (years)	0.992	0.872–1.127	0.898			
Male age (years)	1.049	0.93–1.183	0.439			
Basal FSH (IU/L)	0.962	0.874–1.06	0.438			
Basal LH (IU/L)	0.938	0.728–1.21	0.624			
AFC (n)	0.894	0.659–1.214	0.473			
Protocol of COS	2.074	0.463–9.291	0.34			
Duration of COS (day)	0.928	0.771–1.118	0.431			
Endometrium thickness on hCG trigger day (mm)	0.954	0.726–1.254	0.737			
E2 level on hCG trigger day / Number of oocytes retrieved	1.000	0.999–1.000	0.422			
LH level on hCG trigger day (IU/L)	1.169	1.029–1.327	0.016	1.149	1.007–1.310	0.038
FSH level on hCG trigger day (IU/L)	1.001	0.939–1.067	0.987			
P level on hCG trigger day (ng/ml)	6.658	0.78–56.819	0.083	2.776	0.266–28.921	0.393
Number of 2PN (n)	0.611	0.29–1.287	0.195			
Number of oocytes retrieved/AFC	0.717	0.347–1.482	0.369			

**Notes.**

Data were analyzed using binomial logistic regression. Variables significant in univariate analysis were further included in multivariate analysis. OR, odds ratio; CI, confidence interval. GH(+), growth hormone adjuvant treatment cycle; GH(−), non-GH cycle. Statistical significance was defined as *P*  <  0.05.

## Discussion

In this single-center retrospective cohort study, the results indicated that the GH adjuvant treatment significantly increased the number of oocytes retrieved in POR patients who had no viable embryos in their previous IVF cycles.

As this was a self-controlled study, since the GH+ group received GH adjuvant therapy following a previous cycle with no transferable embryos, both the female patients and their male partners in the GH+ group were significantly older than those in the GH− group. Furthermore, a mean patient age exceeding 40 years was associated with further deterioration of ovarian function, resulting in a reduced number of retrievable oocytes or embryos. However, the results suggested that GH adjuvant treatment improved several variables in the GH+ cycle including the number of 2PN embryos, the P level on the trigger day, and the number of transferable embryos. These findings were consistent with another self-controlled study involving 150 POR patients (Feng et al., 2023), which reported that the GH+ group experienced significant increases in the number of oocytes retrieved, 2PN embryos, transferable embryos, and high-quality embryos. However, unlike that study, the current study did not collect data on high-quality embryos.

To further clarify whether GH adjuvant treatment increased the number of transferable embryos, the study group data was compared with the control group. The results showed that GH adjuvant treatment did not significantly increase the number of transferable embryos, but it did significantly improve the number of oocytes retrieved and was associated with a lower LH level on the trigger day. These findings are consistent with the systematic review and network meta-analysis, which indicate that GH adjuvant therapy increases oocyte yield (Zhang et al., 2020).

Current research suggests that GH plays a crucial role in ovarian function, steroidogenesis, follicular development, and oocyte maturation (Han et al., 2022; Eckery et al., 1997). GH can synergistically stimulate the activity of ovarian aromatase and increase the number of LH receptors on granulosa cells, thereby enhancing the action of Gn and improving the ovarian sensitivity to Gn. Multiple studies have reported that GH adjuvant treatment in POR patients is associated with higher numbers of oocytes retrieved and 2PN embryos (Zhu et al., 2023).

The current study showed that the number of oocytes retrieved from the increased group had a higher LH level on the trigger day in the first cycle. Moreover, GH adjuvant treatment resulted in better outcomes for POR patients with a higher LH level on the previous trigger day. The optimal cut-off value proposed by the ROC analysis for the LH level was 8.755 IU/L, with a sensitivity of 0.765 and specificity of 0.731.

In patients with POR, elevated baseline levels of LH and FSH adversely affect follicular maturity. Specifically, increased LH levels activate multiple signaling pathways in follicular membrane cells, promoting the synthesis and secretion of androgens. However, the activity of aromatase in granulosa cells decreases, making it difficult for androgens to convert to estrogen and E2, leading to excessive androgen levels (Drakopoulos et al., 2020; Liang et al., 2024). This surplus negatively regulates follicular growth. Elevated LH levels also promote the cytotoxic effects of cytokines, inducing apoptosis in granulosa cells and resulting in the atresia of underdeveloped follicles (Santi et al., 2017).

In 2002, scholars introduced the concept of the “LH treatment window”, stipulating that serum LH levels must fall within a specific range to ensure the normal secretion of ovarian steroid hormones and to maintain follicular development in an optimal condition (Shoham, 2002). If LH levels drop below the lower threshold, it could result in inadequate estrogen levels within the follicular fluid, thereby impairing the growth and maturation of the follicle. Conversely, if LH levels exceed the upper threshold, it may trigger an excessive levels of androgens, which in turn suppresses granulosa cell proliferation and aromatase activity, potentially causing follicular atresia or premature luteinization of the pre-ovulatory follicle, ultimately oocyte development (Jabarpour et al., 2024; Wong et al., 2011).

As a result, for the patients with higher LH levels on the previous trigger day, GH may improve the ovarian microenvironment by upregulating its own receptors and enhancing mitochondrial activity, thereby promoting follicular development. Additionally, GH can indirectly stimulate the ovaries through the growth hormone-releasing hormone/GH/IGF-1 axis, which increases the number of gonadotropin receptors and enhances the ovarian responsiveness to Gn (Feng et al., 2023). This regulation of hormone production facilitates follicular development and oocyte maturation. Furthermore, the increase in high-quality follicles raises estrogen levels, which in turn inhibits the secretion of pituitary LH through a negative feedback mechanism. Finally, the moderate decline in LH level might reflect an enhanced sensitivity of follicles to LH due to GH adjuvant treatment, promoting an increase in follicle quantity and consequently raising the number of oocytes retrieved.

This study has several limitations. The retrospective and observational nature of this study presented major obstacles, as the effect of GH might only be associative. The GH+ cycles were conducted following the GH- cycles, indicating that the GH+ cycles had a greater number of preceding IVF attempts, which could result in clinician bias. Another limitation of the present study is the relatively small sample size, which may restrict the statistical power and limit the generalizability of the findings. Additionally, although previous research has suggested that different ovarian stimulation protocols do not independently affect embryo quality, the heterogeneity of stimulation protocols in this study’s retrospective cohort may still introduce potential bias. Variations in Gn dosage, timing, and suppression strategies could interfere with the observed effects of GH adjuvant treatment. Future prospective studies with larger sample sizes and standardized COH protocols are required to minimize protocol-related confounding and to more accurately evaluate the true impact of GH on ovarian response in POR patients.

In conclusion, this study indicated that GH adjuvant treatment increased the number of oocytes retrieved in patients with POR, particularly among those presenting with higher LH levels on the trigger day in previous cycles. These results provide meaningful clinical insight into identifying subgroups of POR patients who may benefit from GH supplementation. While the study data support the potential value of GH in enhancing ovarian response, larger prospective studies with standardized stimulation protocols are needed to further validate these observations and to establish broader clinical recommendations.

##  Supplemental Information

10.7717/peerj.21000/supp-1 Supplemental Information 1 The data on patients with POR.

10.7717/peerj.21000/supp-2 Supplemental Information 2 The explanatory note for the data of patients with POR

## Additional Information and Declarations

### Data Availability

The following information was supplied regarding data availability: The raw measurements are available in the Supplemental Files.
