# Supplementary material for: Effect of growth hormone adjuvant treatment on oocyte retrieval in patients with poor ovarian response and no viable embryos in previous in vitro fertilization (IVF) cycles
Source: PeerJ. 2026 Mar 16;14:e21000. doi: 10.7717/peerj.21000 (PMC13001661; doi:10.7717/peerj.21000)
Supplement: Supplemental Information 2 [file peerj-14-21000-s002.docx]

Note：

In the POR sheet, columns with the suffix “_0” (e.g., xx_0) represent data from the first cycle, while those without this suffix represent data from the second cycle.

In the Ctrl sheet, columns with the suffix “_1” (e.g., xx_1) represent data from the second cycle, while those without this suffix represent data from the first cycle.

The protocols

1 = Progestin Primed Ovarian Stimulation (PPOS) protocol

2 = GnRH agonist long protocol

3 = GnRH antagonist protocol

4 = Mild stimulation protocol
